# Second Law Analysis of Dissipative Nanofluid Flow over a Curved Surface in the Presence of Lorentz Force: Utilization of the Chebyshev–Gauss–Lobatto Spectral Method

**DOI:** 10.3390/nano9020195

**Published:** 2019-02-02

**Authors:** Muhammad Idrees Afridi, Muhammad Qasim, Abderrahim Wakif, Abid Hussanan

**Affiliations:** 1 Department of Mathematics, COMSATS University Islamabad (CUI), Park Road, Tarlai Kalan, Islamabad 455000, Pakistan; idreesafridi313@gmail.com (M.I.A.); mq_qau@yahoo.com (M.Q.); 2 Laboratory of Mechanics, Faculty of Sciences Aïn Chock, Hassan II University, B.P. 5366 Mâarif, Casablanca 20000, Morocco; wakif.abderrahim@gmail.com; 3 Division of Computational Mathematics and Engineering, Institute for Computational Science, Ton Duc Thang University, Ho Chi Minh City 700000, Vietnam; 4 Faculty of Mathematics and Statistics, Ton Duc Thang University, Ho Chi Minh City 700000, Vietnam

**Keywords:** second law analysis, heat transfer, variable thermal conductivity, frictional and Ohmic dissipation, curved surface, nanofluid, Chebyshev–Gauss–Lobatto spectral method

## Abstract

The primary objective of the present work is to study the effects of heat transfer and entropy production in a nanofluid flow over a curved surface. The influences of Lorentz force and magnetic heating caused by the applied uniform magnetic field and energy dissipation by virtue of frictional heating are considered in the problem formulation. The effects of variable thermal conductivity are also encountered in the present model. The dimensional governing equations are reduced to dimensionless form by introducing the similarity transformations. The dimensionless equations are solved numerically by using the Chebyshev–Gauss–Lobatto spectral method (CGLSM). The rate of increase/increase in the local Nusselt number and skin friction coefficient are estimated by using a linear regression model. The expression for dimensionless entropy production is computed by employing the solutions obtained from dimensionless momentum and energy equations. Various graphs are plotted in order to examine the effects of physical flow parameters on velocity, temperature, and entropy production. The increase in skin friction coefficient with magnetic parameter is high for nanofluid containing copper nanoparticles as compared to silver nanoparticles. The analysis reveals that velocity, temperature, and entropy generation decrease with the rising value of dimensionless radius of curvature. Comparative analysis also reveals that the entropy generation during the flow of nanofluid containing copper nanoparticles is greater than that of containing silver nanoparticles.

## 1. Introduction

Boundary layer flow over a stretching surface has extensive applications in industrial products and different engineering processes. The interminable list of its engineering applications includes paper production, a wind-up roll, manufacturing of plastic sheets and metal wires, extrusion of polymer sheets, drawing plastic films, wire drawing, glass blowing metal spinning, cooling thread traveling between a freed roll, and so forth. Sakiadis [1], Fox et al. [2], Tsou et al. [3], Gupta and Gupta [4], Magyari and Keller [5], and Wang et al. [6] are the pioneers of the work on boundary layer flow induced by a stretching surface. Recently, Vajravelu et al. [7] studied the rotating magnetohydrodynamic (MHD) flow over an elastic sheet of variable thickness with Hall and suction/injection effects. Butt et al. [8] studied the influences of magnetic force and internal heat generation on dusty fluid flow over a stretching disk. The parametric study of viscoelastic fluid flow in the presence of thermal radiation, mixed convection, and constant magnetic field is reported by Hsiao [9]. The flow of viscous fluids and nanofluids over a wavy surface, along with heat transfer analysis, are extensively discussed in the book of Shenoy et al. [10]. Recently, Rosca and Pop [11] examined the influence of mass suction on unsteady flow over a curved stretching/shrinking sheet. They used curvilinear coordinates and found multiple solutions using bvp4c. Furthermore, stability analysis was also performed in order to point out the solution which was stable. The influences of Soret and Dufour effects on a flow of nanofluid over a curved surface under the influence of nonlinear thermal radiation were investigated by Reddy et al. [12]. Pop et al. [13] recently reported the impacts of magnetic field on unsteady flow over a curved stretching/shrinking surface. The practical utilization of fluid flow over an elastic curved surface is in stretch-forming machines with curving jaws. 

Nanofluids are fluids which are obtained by dispersing the nanoparticles in a base fluid. The nanoparticles can be made of metals (Cu, Al), carbides (SiC), nonmetals (graphite), carbon nanotubes, and oxides (CuO), while the base fluids may include oil, biofluids, polymer solutions, ethylene glycol, and engine oil. The nanoparticles typically have the dimension of order 10 nm. These nanoparticles are stably suspended in the base fluid unlike in conventional solid–liquid suspensions, and the nanofluids do not cause clogging or abrasion. These exhibits enhanced thermal, magnetic, and electrical properties. The conventional base fluids, like water and oil, etc., have low thermal conductivity and in order to enhance the thermal conductivity of base fluids, nanoparticles are added to base fluids. An ideal nanofluid should possess the highest thermal properties with minimum concentration of nanoparticles in the base fluids. These fluids are basically used for cooling purposes, both at the microlevel, like in electronic chips, and at the macrolevel, like in car engines and jets. Initially, Choi [14] introduced the word “nanofluid” for fluids containing nanoparticles. Recently, Sulochana et al. [15] studied ferrofluid flow over a thin needle under the influence of Lorentz force. They reported that the velocity of Fe_3_O_4_–water is greater than the velocity of Fe_3_O_4_–methanol. Mutuku and Makinde [16] theoretically investigated the effects of double stratification on MHD flow over a flat surface in the presence of mass transfer phenomenon. Khan et al. [17] reported the flow and first law analysis of thin film nanofluid flow sprayed over a stretching cylinder. The flow and thermal analysis of Williamson nanofluid thin film flow with variable fluid properties is investigated by Khan et al. [18]. The effects of variable magnetic field on the three-dimensional flow of linear radiative nanofluid are studied by Nayak et al. [19]. Sheikholeslami and Ganji [20] investigated the Marangoni boundary layer flow of CuO–H_2_O nanofluid in the presence of magnetic fields and absence of viscous dissipation. Das and Jana [21] reported the natural convection flow of nanofluid over a vertical plate by taking the effects of Lorentz force. Stability analysis of mixed convection flow of nanofluid over a permeable cylinder with the effects of radiation and porous medium is performed by Abu Bakar et al. [22]. The stability analysis of nanofluid flow past over a vertical thin needle by taking the effects of mixed convection is reported by Salleh et al. [23]. Soret and Dufour effects on stagnation point flow nanofluid over a stretching/shrinking sheet with stability analysis are studied by Najib et al. [24]. The parametric study of micropolar nanofluid flow in the presence of viscous dissipation and constant magnetic field is reported by Hsiao [25].

Viscous dissipation plays an important role in the heat transfer analysis, especially in boundary layer flows, due to high velocity gradients inside the boundary layer. The energy dissipation acts like a heat source and which is why it leads to an appreciable rise in the fluid temperature. Due to the high velocity gradients inside the boundary layer, the kinetic energy of fluid is converted into thermal energy and enhances the fluid temperature. Gebhart [26], for the first time, pointed out the enhancement of fluid temperature in a natural convection flow. Recently, Afridi and Qasim [27] reported the influence of viscous dissipation on thermal transfer in a nonlinear radiative fluid flow over moving thing needle. Makinde [28] studied the classical Sakiadis flow of nanofluid with Newtonian and frictional heating. The first law analysis of nanofluid flow over a vertical flat surface in the presence of inclined magnetic field and viscous dissipation is reported by Sandeep and Sugunamma [29]. Lin et al. [30] numerically studied the impacts of viscous dissipation on heat transfer in the flow of pseudo-plastic nanofluid thin film with variable thermal conductivity. Ohmic heating and viscous dissipation effects on viscous fluid flow, thermal radiation, velocity, and thermal slip are reported by Sreenivasulu et al. [31]. Some of the recent studies on boundary layer flows are reported in [32,33,34].

The law of thermodynamics declares that all forms of energy are conserved and convertible to another form of energy. The second law of thermodynamics puts a limitation on the conversion of some form of energy to others. The second law indicates that heat cannot be entirely converted into work. That portion of heat which cannot be converted into work is called unavailable energy, and it needs to be rejected as low-grade heat after the work has been done. The availability of energy in a thermal system always decreases, and this unavailability of energy is called entropy. In real thermal processes, the availability of energy decreases, and this phenomenon is called entropy generation. There are different sources that cause entropy generation, particularly in a fluid flow, for example, heat transfer, viscous dissipation, magnetic dissipation, and energy dissipation due to porous medium. Many researchers performed the analysis of entropy generation in boundary layer flows to minimize entropy generation. Recently, Farooq et al. [35] reported the effects of transpiration and viscous dissipation on hybrid nanofluid flow over nonlinear stretching disk. The impacts of variable transport properties on entropy generation in a nonlinear radiative flow over a Riga plate are reported by Afridi et al. [36]. Das et al. [37] investigated the entropy generation in nanofluid flow over a disk with convective boundary condition and porous medium. The entropy generation in a mixed convection flow of nanofluid in a vertical porous channel is investigated by Makinde and Tshehla [38] in the presence of Lorentz force. Some of the recent investigations on the minimization of entropy generation are reported in [39,40,41,42,43,44,45,46,47,48].

In the present study, we reported the impacts of magnetic and viscous dissipation on the heat transfer in a flow of nanofluid over a curved stretching surface. The thermal conductivity of nanofluid is taken to be temperature dependent. In addition, second law analysis is also performed. The reduced momentum and energy equations are solved numerically using the Chebyshev–Gauss–Lobatto spectral method (CGLSM). The transformed set of equations are also solved using the generalized differential quadrature method (GDQM) and Runge–Kutta method. The obtained results are compared and found to be in an excellent agreement. The obtained numerical results are tabulated and discussed comprehensively by plotting against the similarity variable ξ for different values of physical flow parameters.

## 2. Description of the Mathematical Formulation

**Figure 1:**
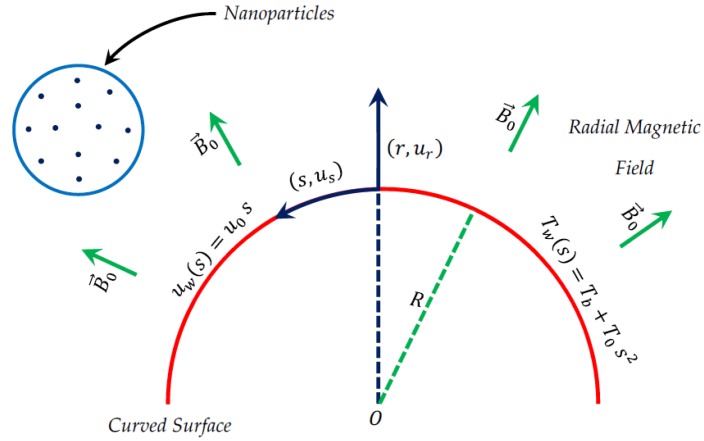
Description of the physical flow model in the curvilinear coordinate system.

As schematically shown in Figure 1, we consider an incompressible flow of nanofluid over a curved surface at r=R with the frictional and Ohmic heating. Moreover, it is presumed that the radial magnetic field ${\vec{B}}_{0}$ is uniform and acting outwardly on the nanofluid flow. Furthermore, the nanofluid thermal conductivity $k_{nf}^{*}$ is taken to be temperature dependent, which is written in the form $k_{nf}^{*}=k_{nf\;}\omega \left(T\right)$, so that $\omega \left(T\right)=1+\varepsilon \left[\left(T-T_{b}\right)/\left(T_{w}-T_{b}\right)\right]$, where ε is a thermal control parameter related to the thermal conductivity. The thermophysical properties of some nanoparticles and water are tabulated in Table 1. Curvilinear coordinates (r,s) are used in the mathematical formulation, where r is normal to any tangent at the curved surface and the coordinate of the arc length s is along the flow direction. The stretching velocity and temperature of the curved sheet are taken to be $u_{w}(s)=u_{o}s$, and $T_{w}(s)=T_{b}+T_{o}s^{2}$, $u_{o}$ is a dimensional constant, $T_{b}$ represents the temperature of the bulk fluid (outside the edge of boundary layer), and $T_{o}$ indicates a dimensional constant.

Under the abovementioned assumptions along with the Prandtl boundary layer approximations, the governing equations of total conservation of mass, momentum, and thermal energy corresponding to the present physical problem can be written in curvilinear coordinates as follows [12]:

(1) $$\frac{\partial }{\partial r}\left(r^{*}u_{r}\right)+R\frac{\partial u_{s}}{\partial s}=0\;,$$

(2) $$\frac{1}{r^{*}}u_{s}^{2}=\frac{1}{\rho_{nf}}\frac{\partial p}{\partial r}\;,$$

(3) $$\rho_{nf}\left(u_{r}\frac{\partial u_{s}}{\partial r}+\frac{R}{r^{*}}u_{s}\frac{\partial u_{s}}{\partial s}+\frac{1}{r^{*}}u_{s}u_{r}\right)=-\frac{R}{r^{*}}\frac{\partial p}{\partial s}+\mu_{nf}\left(\frac{\partial^{2}u_{s}}{\partial r^{2}}+\frac{1}{r^{*}}\frac{\partial u_{s}}{\partial r}-\frac{1}{r^{*}{}^{2}}u_{s}\right)-\sigma_{nf}B_{o}^{2}u_{s}\;,$$

(4) $${\left(\rho c_{p}\right)}_{nf}\left(u_{r}\frac{\partial T}{\partial r}+\frac{R}{r^{*}}u_{s}\frac{\partial T}{\partial s}\right)=\frac{\;k_{nf}}{r^{*}}\frac{\partial }{\partial r}\left(r^{*}\omega \left(T\right)\frac{\partial T}{\partial r}\right)+\mu_{nf}{\left(\frac{\partial u_{s}}{\partial r}-\frac{1}{r^{*}}u_{s}\right)}^{2}+\sigma_{nf}B_{o}^{2}u_{s}^{2}.$$

Here, $r^{*}=r+R$ is the modified space variable.

The boundary layer equations (i.e., Equations (1)–(4)) described the physical model under consideration, subjected to the following boundary conditions [13]:

(5) $$u_{s}=u_{w},\;u_{r}=0,\;T=T_{w}\;\mathrm{at}\;r=0,$$

(6) $$u_{s}\to 0,\;\frac{\partial u_{s}}{\partial r}\to 0,\;T\to T_{b}\;\mathrm{as}\;r\to \infty .$$

Here, $\left(u_{s},u_{r}\right)$ are velocity components in the direction of s and r directions, $B_{o}$ is strength of applied magnetic field, p shows r dependent pressure, T indicates fluid temperature, and $T_{w}$ and $T_{b}$ represent the temperature of curved sheet and fluid in the stress free region, respectively.

According to Das and Jana [21], the expressions of the effective thermophysical properties of the studied nanofluids, like the density $\rho_{nf}$, the heat capacity ${\left(\rho c_{p}\right)}_{nf}$, the dynamic viscosity $\mu_{nf}$, the electric conductivity $\sigma_{nf}$, as well as the dynamic viscosity $k_{nf}$, are given by [17]:

(7) $$\rho_{nf}=\left(1-\phi \right)\rho_{bf}+\phi \rho_{s},\;$$

(8) $${\left(\rho c_{p}\right)}_{nf}=\left(1-\phi \right){\left(\rho c_{p}\right)}_{bf}+\phi {\left(\rho c_{p}\right)}_{s},$$

(9) $$\mu_{nf}=\frac{\mu_{bf}}{{\left(1-\phi \right)}^{2.5}},$$

(10) $$\frac{\sigma_{nf}}{\sigma_{bf}}=1+\frac{3\left(\frac{\sigma_{s}}{\sigma_{bf}}-1\right)\phi }{\left(\frac{\sigma_{s}}{\sigma_{bf}}+2\right)-\left(\frac{\sigma_{s}}{\sigma_{bf}}-1\right)\phi },\;$$

(11) $$\frac{k_{nf}}{k_{bf}}=1+\frac{3\left(\frac{k_{s}}{k_{bf}}-1\right)\phi }{\left(\frac{k_{s}}{k_{bf}}+2\right)-\left(\frac{k_{s}}{k_{bf}}-1\right)\phi }.\;$$

Here, ϕ represents the nanoparticles solid volume fraction, where the subscripts bf and s show the base fluid and the solid nanoparticles, respectively.

Due to the interesting thermophysical properties of metallic nanoparticles and their great potential applications in nanotechnology, we chose to use silver Ag and copper Cu nanoparticles among those kind of engineered particles as the dispersed solid phase in a specified base fluid (e.g., water) for obtaining Ag–water and Cu–water nanofluids, whose thermophysical properties of their constituents are clearly outlined in Table 1.

In order to get the dimensionless form of the governing partial differential equations, it is more suitable in this investigation to use the following similarity transformations [11]:

(12) $$\xi ={\left(\frac{u_{o}}{\nu_{bf}}\right)}^{0.5}r,\;g^{\prime }\left(\xi \right)=\frac{u_{s}\left(r,s\right)}{u_{w}},\;g\left(\xi \right)=-\frac{1}{{\left(u_{o}\nu_{bf}\right)}^{0.5}}\left(\frac{\;r^{*}}{R}\right)u_{r}\left(r,s\right),\;\;\theta =\frac{T-T_{b}}{T_{w}-T_{b}},\;P\left(\xi \right)=\frac{1}{\rho_{bf}u_{o}^{2}s^{2}}p,\;\kappa =R{\left(\frac{u_{o}}{\nu_{bf}}\right)}^{0.5},$$

where κ denotes the curvature parameter.

By substituting the dimensionless variables shown above into Equations (1)–(4), and putting

(13) $$A_{1}=\frac{\sigma_{nf}}{\sigma_{bf}},\;A_{2}={\left(1-\phi \right)}^{2.5},\;A_{3}=\left(1-\phi \right)+\phi \left(\frac{\rho_{s}}{\rho_{bf}}\right),\;A_{4}=\left(1-\phi \right)+\phi \frac{{\left(\rho c_{p}\right)}_{s}\;}{{\left(\rho c_{p}\right)}_{bf}},\;A_{5}=\frac{k_{nf}}{k_{bf}},$$

We obtain, after some rearrangement,

(14) $$\frac{\partial P}{\partial \xi }=\frac{A_{3}}{h\;}{g^{\prime }}^{2},$$

(15) $$\frac{2\kappa }{A_{3}h}P=\frac{1}{A_{2}A_{3}}\left(g^{\prime \prime \prime }+\frac{g^{\prime \prime }}{h}-\frac{g^{\prime }}{h^{2}}\right)+\frac{\kappa }{h}gg^{\prime \prime }+\frac{\kappa }{h^{2}}gg^{\prime }-\frac{\kappa }{h}{g^{\prime }}^{2}-\frac{A_{1}M}{A_{3}}g^{\prime },$$

(16) $$\frac{A_{5}}{A_{4}\mathrm{Pr}}\left(1+\varepsilon \theta \right)\left({\theta^{\prime }}^{\prime }+\frac{\theta^{\prime }}{h}\right)+\frac{\varepsilon A_{5}}{A_{4}\mathrm{Pr}}{\theta^{\prime }}^{2}+\left[\begin{array}{l} \frac{\kappa }{h}\left(g\theta^{\prime }-2g^{\prime }\theta \right)+\frac{Ec}{A_{2}A_{4}}{\left({g^{\prime }}^{\prime }-\frac{g^{\prime }}{h}\right)}^{2}+\\ \frac{A_{1}EcM}{A_{4}}{g^{\prime }}^{2} \end{array}\right]=0\;,$$

Here, the prime denotes the derivative with respect to ξ and

(17) h(ξ)=ξ+κ .

Accordingly, the boundary conditions given by Equations (5) and (6) are then written in dimensionless form as follows:

(18) $$g\left(0\right)=0,\;g^{\prime }\left(0\right)=1,\;\theta \left(0\right)=1,$$

(19) $$g^{\prime }\left(\xi \to \infty \right)\to 0,\;g^{\prime \prime }\left(\xi \to \infty \right)\to 0,\;\theta \left(\xi \to \infty \right)\to 0.$$

Furthermore, the different dimensionless parameters shown in Equations (15) and (16) are defined as

(20) $$Ec=\frac{\rho_{bf}u_{w}^{2}}{{\left(\rho c_{p}\right)}_{bf}\left(T_{w}-T_{b}\right)}\;(\mathrm{Eckert}\;\mathrm{number}),\;\;M=\frac{B_{o}^{2}\sigma_{bf}}{u_{o}\rho_{bf}}\;(\mathrm{magnetic}\;\mathrm{parameter}),\;\;\mathrm{Pr}=\frac{\nu_{bf}}{\alpha_{bf}}\;(\mathrm{Prandtl}\;\mathrm{number}).$$

Here, $\nu_{bf}$ and $\alpha_{bf}$ are the thermal diffusivity and kinematic viscosity of the base fluid, respectively.

After derivation of Equation (15) with respect to ξ, the pressure gradient term ∂P/∂ξ appearing in the resulting equation can be eliminated and replaced by its expression $A_{3\;}{g^{\prime }}^{2}/h$ shown in Equation (14). Hence, the dimensionless momentum equation becomes

(21) $$g^{\prime \prime \prime \prime }+\frac{2}{h}g^{\prime \prime \prime }-g_{1}g^{\prime \prime }+g_{2}g^{\prime }+\frac{A_{2}A_{3}\kappa }{h}\left(gg^{\prime \prime \prime }+\frac{g}{h}g^{\prime \prime }-\frac{1}{h}{g^{\prime }}^{2}-g^{\prime }g^{\prime \prime }-\frac{g}{h^{2}}g^{\prime }\right)=0,$$

where

(22) $$g_{1}=\frac{1\;}{h^{2}}+A_{1}A_{2}M,$$

(23) $$g_{2}=\frac{1\;}{h^{3}}-\frac{A_{1}A_{2}M}{h}.$$

From the engineering point of view, the very important physical quantities of interest are the local skin friction coefficient $Cf_{s}$ and the local Nusselt number $Nu_{s}$, which are given formally by

(24) Res0.5Cfs=1A2(g″(0)−1κg′(0)),

(25) Res−0.5Nus=−A5(θ′(0)+εθ′2(0)).

Here, Res(=uws/νbf) represents the local Reynolds number. 

Upon making use of the following key transformations

(26) $$\left\{ \begin{array}{l} \xi =f\left(\eta \right),\\ g\left(\xi \right)=g\left(f\left(\eta \right)\right)=G\left(\eta \right),\\ h\left(\xi \right)=h\left(f\left(\eta \right)\right)=H\left(\eta \right),\\ \theta \left(\xi \right)=\theta \left(f\left(\eta \right)\right)=\Theta \left(\eta \right), \end{array}\right.$$

Equations (16) and (21) with the boundary condition (18) and (19) can be rewritten in the form

(27) $$L_{G}\left(G\right)+N_{G}\left(G,\Theta \right)\;=0,\;$$

(28) $$L_{\Theta }\left(\Theta \right)+N_{\Theta }\left(G,\Theta \right)\;=0,\;$$

(29) $$G\left(\eta \right)=0,\;G^{\prime }\left(\eta \right)=\frac{\xi_{\infty }}{2},\;\Theta \left(\eta \right)=1,\;\mathrm{at}\;\eta =-1,$$

(30) $$G^{\prime }\left(\eta \right)\to 0,\;{G^{\prime }}^{\prime }\left(\eta \right)\to 0,\;\Theta \left(\eta \right)\to 0,\;\mathrm{as}\;\eta \to 1.$$

Here,

(31) $$f\left(\eta \right)=\frac{\xi_{\infty }\left(\eta +1\right)}{2}\;,$$

(32) $$\left\{ \begin{array}{l} g^{\left(n\right)}\left(\xi \right)={\left(\frac{2}{\xi_{\infty }}\right)}^{n}G^{\left(n\right)}\left(\eta \right),\\ \theta^{\left(n\right)}\left(\xi \right)={\left(\frac{2}{\xi_{\infty }}\right)}^{n}\Theta^{\left(n\right)}\left(\eta \right), \end{array}\right.$$

where $\xi_{\infty }$ is the asymptotic value of the boundary layer thickness and n denotes the integer-order derivative with respect to the spatial variables ξ (i.e., for g and θ or η (i.e., for G and Θ). In view of Equations (27) and (28), the linear and nonlinear parts $L_{G}\left(G\right)$, $L_{\Theta }\left(G\right)$, $N_{G}\left(G,\Theta \right)$ and $N_{\Theta }\left(G,\Theta \right)$, arising from Equations (16) and (21), are expressed explicitly as follows:

(33) $$L_{G}\left(G\right)=\;G^{\prime \prime \prime \prime }+\frac{\xi_{\infty }}{H}G^{\prime \prime \prime }-\frac{\xi_{\infty }^{2}}{4}\left(\frac{1\;}{H^{2}}+A_{1}A_{2}M\right)G^{\prime \prime }+\frac{\xi_{\infty }^{3}}{8H}\left(\frac{1\;}{H^{2}}-A_{1}A_{2}M\right)G^{\prime },$$

(34) $$L_{\Theta }\left(\Theta \right)=\frac{\xi_{\infty }^{2}A_{5}}{4A_{4}\mathrm{Pr}}\Theta^{\prime \prime }+\frac{\xi_{\infty }^{3}A_{5}}{8A_{4}\mathrm{Pr}H}\Theta^{\prime },$$

(35) $$N_{G}\left(G,\Theta \right)=\frac{\xi_{\infty }A_{2}A_{3}\kappa }{2H}\left(GG^{\prime \prime \prime }+\frac{\xi_{\infty }G}{2H}G^{\prime \prime }-\frac{\xi_{\infty }}{2H}{G^{\prime }}^{2}-G^{\prime }G^{\prime \prime }-\frac{\xi_{\infty }^{2}G}{4H^{2}}G^{\prime }\right),$$

(36) $$N_{\Theta }\left(G,\Theta \right)=\left\{\begin{array}{l} \frac{\xi_{\infty }^{2}\varepsilon A_{5}}{4A_{4}\mathrm{Pr}}{\Theta^{\prime }}^{2}+\frac{\xi_{\infty }^{3}\varepsilon A_{5}\Theta }{8A_{4}\mathrm{Pr}H}\Theta^{\prime }+\frac{\xi_{\infty }^{2}\varepsilon A_{5}}{4A_{4}\mathrm{Pr}}\Theta \Theta^{\prime \prime }+\frac{\xi_{\infty }^{3}\kappa_{}G}{8H}\Theta^{\prime }-\frac{\xi_{\infty }^{3}\kappa_{}\Theta }{4H}G^{\prime }+\\ \frac{Ec}{A_{2}A_{4}}{G^{\prime \prime }}^{2}-\frac{\xi_{\infty }Ec}{A_{2}A_{4}H}G^{\prime }G^{\prime \prime }+\frac{\xi_{\infty }^{2}Ec}{4}\left(\frac{1}{A_{2}A_{4}H^{2}}+\frac{A_{1}}{A_{4}}M\right){G^{\prime }}^{2} \end{array}\right\}.$$

By virtue of the transformations considered in Equation (26), the engineering quantities Res0.5Cfs and Res−0.5Nus can be reduced in the following dimensionless form

(37) Res0.5Cfs=2ξ∞A2(2 ξ∞G″(−1)−1κG′(−1)),

(38) Res−0.5Nus=−2A5ξ∞(Θ′(−1)+2εξ∞Θ′2(−1)).

## 3. Analysis of Entropy Production

The expression for entropy generation ${{\dot{E}}^{\prime \prime \prime }}_{G}$ in a nanofluid flow over a curved shape surface by incorporating the effects of variable thermal conductivity, and frictional and Ohmic heating, takes the following form [51]:

(39) $${{\dot{E}}^{\prime \prime \prime }}_{G}={{\dot{E}}^{\prime \prime \prime }}_{GT}+{{\dot{E}}^{\prime \prime \prime }}_{GF}+{{\dot{E}}^{\prime \prime \prime }}_{GM}.$$

Here, ${{\dot{E}}^{\prime \prime \prime }}_{GT}$ shows the entropy production by virtue of heat transfer, ${{\dot{E}}^{\prime \prime \prime }}_{GF}$ represents the entropy production by virtue of frictional heating, and ${{\dot{E}}^{\prime \prime \prime }}_{GM}$ characterizes the contribution of the magnetic field, where [51]

(40) $${{\dot{E}}^{\prime \prime \prime }}_{GT}=k_{nf}\frac{\omega \left(T\right)}{T^{2}}{\left(\frac{\partial T}{\partial r}\right)}^{2},$$

(41) $${{\dot{E}}^{\prime \prime \prime }}_{GF}=\frac{\mu_{nf}}{T}{\left(\frac{\partial u_{s}}{\partial r}-\frac{u_{s}}{r^{*}}\right)}^{2},$$

(42) $${{\dot{E}}^{\prime \prime \prime }}_{GM}=\frac{\sigma_{nf}B_{o}^{2}}{T}u_{s}^{2}.$$

By considering the following characteristic entropy generation

(43) $${{\dot{E}}^{\prime \prime \prime }}_{GC}=\frac{k_{bf}u_{o}}{\nu_{bf}},$$

then, the dimensionless form of entropy generation Ns takes the following form:

(44) $$N_{s}=\frac{{{\dot{E}}^{\prime \prime \prime }}_{G}}{{{\dot{E}}^{\prime \prime \prime }}_{GC}}=\underset{Thermal\;\mathrm{contribution}}{\underset{︸}{A_{5}\frac{\left(1+\varepsilon \theta \right){\theta^{\prime }}^{2}}{{\left(\theta +\lambda \right)}^{2}}}}+\underset{Frictional\;contribution}{\underset{︸}{\frac{Ec\mathrm{Pr}}{A_{2}}\frac{{\left(g^{\prime \prime }-\frac{g^{\prime }}{h\left(\xi \right)}\right)}^{2}}{\left(\theta +\lambda \right)}}}+\underset{Magnetic\;contribution}{\underset{︸}{A_{1}MEc\mathrm{Pr}\frac{{g^{\prime }}^{2}}{\left(\theta +\lambda \right)}}},$$

where $\lambda =T_{b}/\left(T_{w}-T_{b}\right)$ denotes the temperature difference parameter.

By applying the previously recommended transformations, the entropy generation $N_{s}$ becomes 

(45) $$N_{s}=\frac{4A_{5}\left(1+\varepsilon \Theta \right){\Theta^{\prime }}^{2}}{\xi_{\infty }^{2}{\left(\Theta +\lambda \right)}^{2}}+\frac{Ec\mathrm{Pr}}{\xi_{\infty }^{4}A_{2}\left(\Theta +\lambda \right)}{\left(4G^{\prime \prime }-2\xi_{\infty }\frac{G^{\prime }}{H\;}\right)}^{2}+\frac{4A_{1}MEc\mathrm{Pr}{G^{\prime }}^{2}}{\xi_{\infty }^{2}\left(\Theta +\lambda \right)}.$$

## 4. Solution Methodology 

As demonstrated in the last section, the physical model proposed in this investigation, for studying the boundary layer flow of a nanofluid, is described by a set of nonlinear ordinary differential equations (i.e., Equations (27) and (28)). Indeed, as we have already mentioned, the resulting ordinary differential equations (ODEs) can be regarded, from the mathematical point of view, as a highly nonlinear system, and closed form solutions of this problem are almost impossible, except in certain limiting cases, in which the problem can be solved analytically using a suitable method.

Additionally, the studied problem can be handled analytically for the case where ϕ=ε=0 and κ=∞ by employing the Laplace transform and using the confluent hypergeometric function. In this special limiting case, the exact solutions for g(ξ) and θ(ξ) are expressed formally as follows [52]:

(46) $$g\left(\xi \right)=\frac{1-exp\left(-\beta \xi \right)}{\beta },$$

(47) $$\theta \left(\xi \right)=-nW^{2}\left(\xi \right)+\frac{W^{1-m}\left(\xi \right)\left(1+nW^{2}\left(0\right)\right)F_{1,1}\left(-\left(m+1\right);2-m;{-}_{}W\left(\xi \right)\right)}{W^{1-m}\left(0\right)F_{1,1}\left(-\left(m+1\right);2-m;{-}_{}W\left(0\right)\right)},$$

where

(48) $$\beta ={\left(1+M\right)}^{0.5},$$

(49) $$W\left(\xi \right)=\frac{\mathrm{Pr}exp\left(-\beta \xi \right)}{\beta^{2}},$$

(50) $$n=\frac{Ec{\left(2M+1\right)}_{}}{2\left(2{-}_{}W\left(0\right)\right)W\left(0\right)},$$

(51) $$m=1{-}_{}W\left(0\right).$$

Furthermore, the integral form of the Kummer confluent hypergeometric function $F_{1,1}$ used above is defined as

(52) $$F_{1,1}\left(a;b;z\right)=\frac{\Gamma \left(b\right)}{\Gamma \left(b-a\right)\Gamma \left(a\right)}\int_{0}^{1}t^{a-1}{\left(1-t\right)}^{b-a-1}exp{\left(zt\right)}_{}dt.$$

Here, Γ represents the Gamma function, where

(53) $$\Gamma \left(x\right)=\int_{0}^{\infty }t^{x-1}exp{\left({-}_{}t\right)}_{}dt.$$

From the physical point of view, the curvature shape of the scratched surface (i.e.,κ≠∞) and the linear temperature dependence of the thermal conductivity (i.e., ε≠0) introduce further nonlinear terms in the governing equation of the problem, which made it more complex to be investigated analytically, in order to obtain closed form solutions as those described previously in Equations (46) and (47). Consequently, for reducing the complexity encountered in this problem, it is more useful to adopt a suitable numerical procedure to construct the solutions of Equations (27) and (28). To achieve this objective, the resulting ODEs are implemented numerically by discretizing the present boundary layer equations using the Chebyshev–Gauss–Lobatto spectral method (CGLSM), which was developed and well explained by Trefethen [53] and Canuto et al. [54], based on the Chebyshev polynomial interpolation and the following non-uniform grid points

(54) $$\eta_{i}=cos\left(\frac{\pi i-\pi }{N-1}\right).$$

Here, 1≤i≤N and $\eta_{N}\leq \eta_{i}\leq \eta_{1}$, where $\eta_{1}=1$ and $\eta_{N}=-1$.

Following this numerical method, the derivatives of the functions G(η) and Θ(η) with respect to the variable η at a collocation point $\eta_{i}$ are given by

(55) $$\left\{ \begin{array}{l} G^{\left(n\right)}\left(\eta_{i}\right)=\sum_{j=1}^{N}d_{ij}^{\left(n\right)}G\left(\eta_{j}\right)=\sum_{j=1}^{N}d_{ij}^{\left(n\right)}G_{j}\;,\\ \Theta^{\left(n\right)}\left(\eta_{i}\right)=\;\sum_{j=1}^{N}d_{ij}^{\left(n\right)}\Theta \left(\eta_{j}\right)=\sum_{j=1}^{N}d_{ij}^{\left(n\right)}\Theta_{j}\;. \end{array}\right.$$

Here, $d_{ij}^{\left(n\right)}$ are the elements of the $n^{th}$-order Chebyshev differentiation matrix and N is the total number of collocation points considered in this investigation, where i and j are integers varying from 1 to N.

According to Canuto et al. [54], the elements $d_{ij}^{\left(1\right)}$ of the first-order Chebyshev differentiation matrix are given by

(56) $$d_{ij}^{\left(1\right)}=\left\{ \begin{array}{l} \frac{2N^{2}-4N+3}{6},\;\mathrm{for}\;i=j=1,\\ \frac{\eta_{i}}{2\left(\eta_{i}^{2}-1\right)},\;\mathrm{for}\;i=j\neq 1,\\ \frac{{\left(-1\right)}^{i+j}c_{i}}{c_{j}\left(\eta_{i}-\eta_{j}\right)},\;\mathrm{for}\;i\neq j,\\ \frac{-2N^{2}+4N-3}{6},\;\mathrm{for}\;i=j=N, \end{array}\right.$$

where

(57) $$c_{i}=\{\begin{array}{c} 2\;,\;\mathrm{for}\;i=1\;,\;N,\\ 1\;,\;\mathrm{for}\;i\neq 1\;,\;N. \end{array}$$

In addition, the other elements $d_{ij}^{\left(n\right)}$ corresponding to the $n^{th}$-order Chebyshev differentiation matrix are computed using the following recurrence relation

(58) $$d_{ij}^{\left(n\right)}=\overset{k=N}{\underset{k=1}{\sum }}d_{ik}^{\left(n-1\right)}d_{kj}.$$

Here, 1≤i,j≤N and n≥2.

Therefore, after discretization of the studied problem, the dimensionless modified unknowns G(η) and Θ(η) are accurately approximated in each collocation point $\eta_{i}$ by $G_{i}$ $\left(i.e.\;G\left(\eta_{i}\right)\right)$ and $\Theta_{i}$ $\left(i.e.\;\Theta \left(\eta_{i}\right)\right)$, respectively. According to Wakif et al. [49], the discretized form of Equations (27) and (28), together with the boundary conditions (29) and (30), are written as follows

(59) $$\left(S\right):\{\begin{array}{l} \sum_{j=1}^{N}d_{1j}^{\left(1\right)}G_{j}=0,\\ \sum_{j=1}^{N}d_{1j}^{\left(2\right)}G_{j}=0,\\ L_{G_{i}}\left(G_{i}\right)+N_{G_{i}}\left(G_{i},\Theta_{i}\right)\;=0\;,\;\mathrm{for}\;3\leq i\leq N-2,\;\\ G_{N}=0,\\ \sum_{j=1}^{N}d_{Nj}^{\left(1\right)}G_{j}-\frac{\xi_{\infty }}{2}=0,\\ \Theta_{1}=0,\\ L_{\Theta_{i}}\left(\Theta_{i}\right)+N_{\Theta_{i}}\left(G_{i},\Theta_{i}\right)\;=0\;,\;\mathrm{for}\;2\leq i\leq N-1,\;\\ \Theta_{N}-1=0. \end{array}$$

Here,

(60) $$L_{G_{i}}\left(G_{i}\right)=\left\{\begin{array}{l} \left(\sum_{j=1}^{N}d_{ij}^{\left(4\right)}G_{j}\right)+\frac{\xi_{\infty }}{H_{i}}\left(\sum_{j=1}^{N}d_{ij}^{\left(3\right)}G_{j}\right)-\frac{\xi_{\infty }^{2}}{4}\left(\frac{1\;}{H_{i}^{2}}+A_{1}A_{2}M\right)\left(\sum_{j=1}^{N}d_{ij}^{\left(2\right)}G_{j}\right)+\\ \frac{\xi_{\infty }^{3}}{8H_{i}}\left(\frac{1\;}{H_{i}^{2}}-A_{1}A_{2}M\right)\left(\sum_{j=1}^{N}d_{ij}^{\left(1\right)}G_{j}\;\right) \end{array}\right\}\;,$$

(61) $$L_{\Theta_{i}}\left(\Theta_{i}\right)=\frac{\xi_{\infty }^{2}A_{5}}{4A_{4}\mathrm{Pr}}\left(\sum_{j=1}^{N}d_{ij}^{\left(2\right)}\Theta_{j}\right)+\frac{\xi_{\infty }^{3}A_{5}}{8A_{4}\mathrm{Pr}H_{i}}\left(\sum_{j=1}^{N}d_{ij}^{\left(1\right)}\Theta_{j}\right),$$

(62) $$N_{G_{i}}\left(G_{i},\Theta_{i}\right)=\frac{\xi_{\infty }\kappa A_{2}A_{3}}{2H_{i}}\left\{\begin{array}{l} G_{i}\left(\sum_{j=1}^{N}d_{ij}^{\left(3\right)}G_{j}\right)+\frac{\xi_{\infty }G_{i}}{2H_{i}}\left(\sum_{j=1}^{N}d_{ij}^{\left(2\right)}G_{j}\right)-\\ \frac{\xi_{\infty }}{2H_{i}}\left(\sum_{j=1}^{N}d_{ij}^{\left(1\right)}G_{j}\right)\left(\sum_{j=1}^{N}d_{ij}^{\left(1\right)}G_{j}\right)-\\ \left(\sum_{j=1}^{N}d_{ij}^{\left(1\right)}G_{j}\right)\left(\sum_{j=1}^{N}d_{ij}^{\left(2\right)}G_{j}\right)-\frac{\xi_{\infty }^{2}G_{i}}{4H_{i}^{2}}\left(\sum_{j=1}^{N}d_{ij}^{\left(1\right)}G_{j}\right) \end{array}\right\},$$

(63) $$N_{\Theta_{i}}\left(G_{i},\Theta_{i}\right)=\left\{\begin{array}{l} \frac{\xi_{\infty }^{2}\varepsilon A_{5}}{4A_{4}\mathrm{Pr}}\left(\sum_{j=1}^{N}d_{ij}^{\left(1\right)}\Theta_{j}\right)\left(\sum_{j=1}^{N}d_{ij}^{\left(1\right)}\Theta_{j}\right)+\frac{\xi_{\infty }^{3}\varepsilon A_{5}\Theta_{i}}{8A_{4}\mathrm{Pr}H_{i}}\left(\sum_{j=1}^{N}d_{ij}^{\left(1\right)}\Theta_{j}\right)+\\ \frac{\xi_{\infty }^{2}\varepsilon A_{5}}{4A_{4}\mathrm{Pr}}\Theta_{i}\left(\sum_{j=1}^{N}d_{ij}^{\left(2\right)}\Theta_{j}\right)+\frac{\xi_{\infty }^{3}\kappa_{}G_{i}}{8H_{i}}\left(\sum_{j=1}^{N}d_{ij}^{\left(1\right)}\Theta_{j}\right)-\\ \frac{\xi_{\infty }^{3}\kappa_{}\Theta_{i}}{4H_{i}}\left(\sum_{j=1}^{N}d_{ij}^{\left(1\right)}G_{j}\right)+\frac{Ec}{A_{2}A_{4}}\left(\sum_{j=1}^{N}d_{ij}^{\left(2\right)}G_{j}\right)\left(\sum_{j=1}^{N}d_{ij}^{\left(2\right)}G_{j}\right)-\\ \frac{\xi_{\infty }Ec}{A_{2}A_{4}H_{i}}\left(\sum_{j=1}^{N}d_{ij}^{\left(1\right)}G_{j}\right)\left(\sum_{j=1}^{N}d_{ij}^{\left(2\right)}G_{j}\right)+\\ \frac{\xi_{\infty }^{2}Ec}{4}\left(\frac{1}{A_{2}A_{4}H_{i}^{2}}+\frac{A_{1}}{A_{4}}M\right)\left(\sum_{j=1}^{N}d_{ij}^{\left(1\right)}G_{j}\right)\left(\sum_{j=1}^{N}d_{ij}^{\left(1\right)}G_{j}\right) \end{array}\right\},$$

where

(64) $$H_{i}=\left(\kappa +\frac{\xi_{\infty }}{2}\right)+\frac{\xi_{\infty }}{2}cos\left(\frac{\pi i-\pi }{N-1}\right)\;.$$

Finally, the dimensionless governing equations with their associated boundary conditions (i.e., Equations (27)–(30)) have been transformed into an algebraic nonlinear system (S) of 2N equations, which are solved iteratively using the Newton—Raphson method to obtain more precise results, in which the numerical results are given with an absolute accuracy of the order of $10^{-8}$.

Consequently, after computing the accurate discrete set of solutions $\left\{\left(G_{i},\Theta_{i}\right)/1\leq i\leq N\right\}$, the dimensionless quantities Res0.5Cfs and Res−0.5Nus, shown in Equations (24) and (25), can be deduced numerically as follows

(65) Res0.5Cfs=4ξ∞2A2(∑j=1NdNj(2)Gj)−2ξ∞A2κ(∑j=1NdNj(1)Gj),

(66) Res−0.5Nus=−2A5ξ∞(∑j=1NdNj(1)Θj)−4A5εξ∞2(∑j=1NdNj(1)Θj)(∑j=1NdNj(1)Θj).

Under the above key considerations, the entropy generation $N_{s}$, shown in Equation (45), can be computed at each point $\eta_{i}$ by the following formula 

(67) $$N_{s}\left(\eta_{i}\right)=\left\{\begin{array}{l} \frac{4A_{5}\left(1+\varepsilon \Theta_{i}\right)}{\xi_{\infty }^{2}{\left(\Theta_{i}+\lambda \right)}^{2}}\left(\sum_{j=1}^{N}d_{ij}^{\left(1\right)}\Theta_{j}\right)\left(\sum_{j=1}^{N}d_{ij}^{\left(1\right)}\Theta_{j}\right)+\\ \frac{Ec\mathrm{Pr}}{\xi_{\infty }^{4}A_{2}\left(\Theta_{i}+\lambda \right)}{\left[4\left(\sum_{j=1}^{N}d_{ij}^{\left(2\right)}G_{j}\right)-\frac{2\xi_{\infty }}{H_{i}\;}\left(\sum_{j=1}^{N}d_{ij}^{\left(1\right)}G_{j}\right)\right]}^{2}+\\ \frac{4A_{1}MEc\mathrm{Pr}}{\xi_{\infty }^{2}\left(\Theta_{i}+\lambda \right)}\left(\sum_{j=1}^{N}d_{ij}^{\left(1\right)}G_{j}\right)\left(\sum_{j=1}^{N}d_{ij}^{\left(1\right)}G_{j}\right) \end{array}\right\}.$$

## 5. Analysis of Results

The combined effects of variable thermal conductivity and magnetic dissipation on nanofluid heat transfer enhancement and entropy generation in a dissipative medium are performed numerically in this investigation for water-based nanofluids with silver Ag and copper Cu nanoparticles (i.e., Newtonian metallic nanofluids). As mentioned previously in this paper, the exact solutions of the reduced set of governing equations with the considered boundary conditions are not possible due to the high nonlinearity of the studied boundary layer flow problem. Therefore, the numerical scheme knows as Chebyshev–Gauss–Lobatto spectral method (CGLSM) were used carefully to simulate the present problem, in order to obtain very good approximate numerical results in terms of accuracy and computational cost. For this purpose, several numerical tests were carried out in this analysis, in which our findings are clearly illustrated in Table 2, Table 3 and Table 4, in order to show the validity and the efficiency of our numerical code via various limiting cases.

In order to check the exactness of our numerical results, we carried out a self-validation of the present numerical results by computing the engineering quantities −Res0.5Cfs and Res−0.5Nus numerically using Chebyshev–Gauss–Lobatto spectral method (CGLSM), and analytically through Equations (46) and (47), as shown in Table 2 and Table 3, for the case where ϕ=ε=0 and κ=∞. As expected, it is observed from Table 2 and Table 3 that there is a remarkable equality between our numerical and analytical findings, indicating the validity of our numerical implementation. Moreover, to confirm the computational efficiency of the present proposed numerical method, we performed various numerical comparisons for the values of the engineering quantity −Res0.5Cfs, obtained numerically by Chebyshev–Gauss–Lobatto spectral method (CGLSM), generalized differential quadrature method (GDQM) [55,56,57], and Runge–Kutta–Fehlberg method (RKFM) [16,38], as displayed in Table 4 for the case where ϕ=ε=Ec=M=0. A thorough quantitative examination of these numerical results shows, clearly, that there is an excellent agreement among the numerical values obtained by using three numerical methods. Table 5 shows the comparison of numerical values of the skin friction coefficients with the existing results in the literature, in which excellent agreement was found. Hence, the extensive numerical simulation that we have performed demonstrates that the proposed numerical procedure is powerful, and our numerical results are highly accurate. Furthermore, to reach the required absolute accuracy, we took, as key technical parameters, ξ=10 and N=70 for all subsequent analyses.

**Figure 2:**
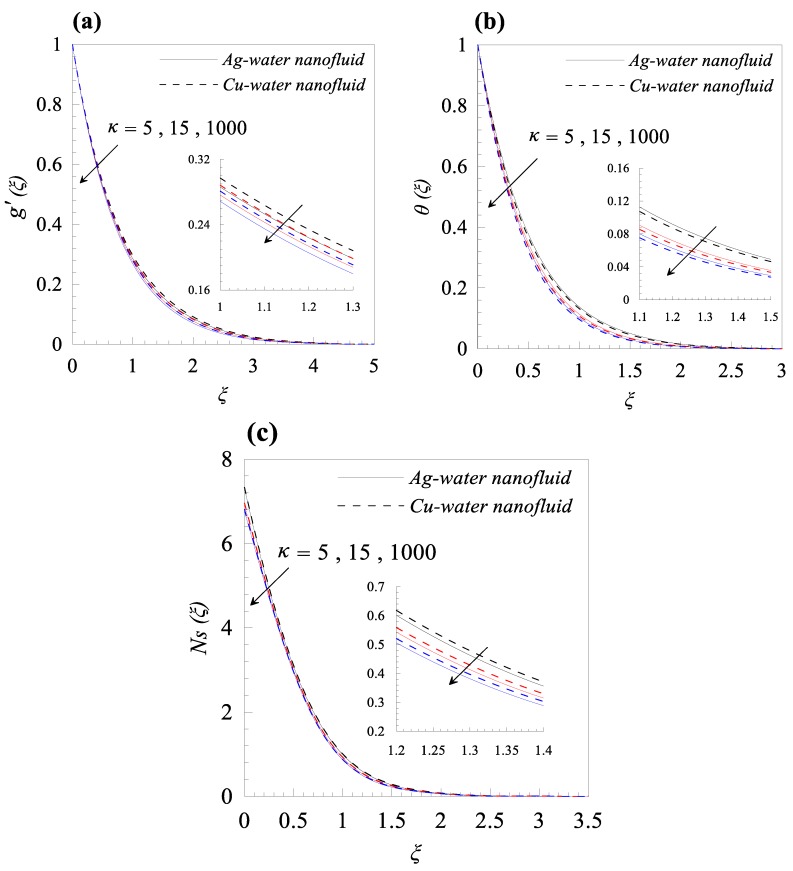
Effects of curvature parameter *κ* on (**a**) velocity profile *g*’(*ξ*), (**b**) temperature distribution *θ*(*ξ*), and (**c**) entropy generation *Ns*(*ξ*), when *M* = 0.2, *φ* = 0.01, *ε* = 0.2, *Ec* = 0.3, and *λ* = 0.5.

**Figure 3:**
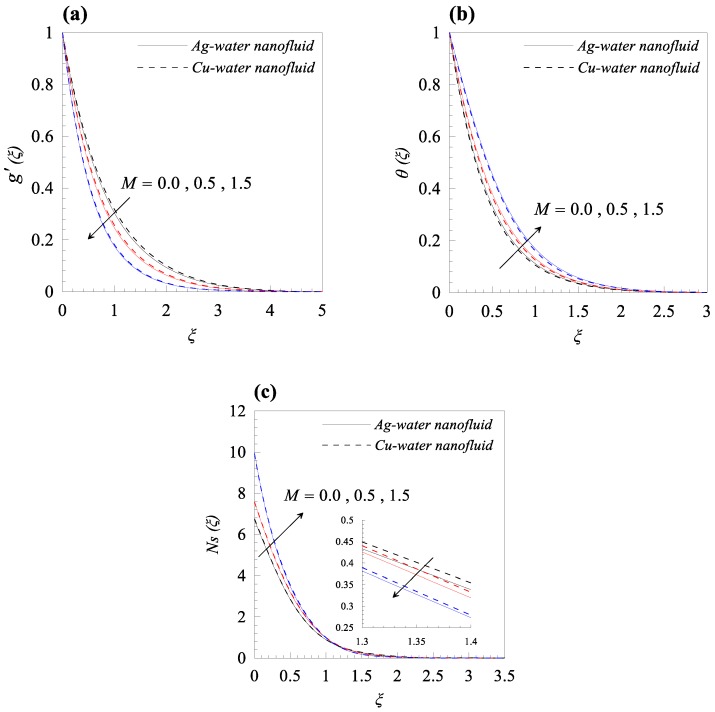
Effects of magnetic parameter *M* on (**a**) velocity profile *g*’(*ξ*), (**b**) temperature distribution *θ*(*ξ*), and (**c**) entropy generation *Ns*(*ξ*), when *κ* = 10, *φ* = 0.01, *ε* = 0.2, *Ec* = 0.3, and *λ* = 0.5.

**Figure 4:**
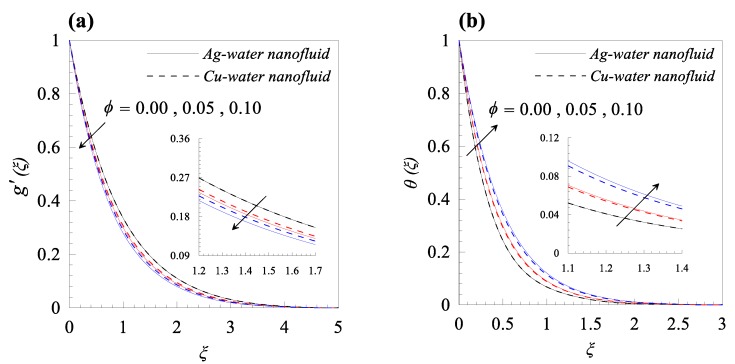

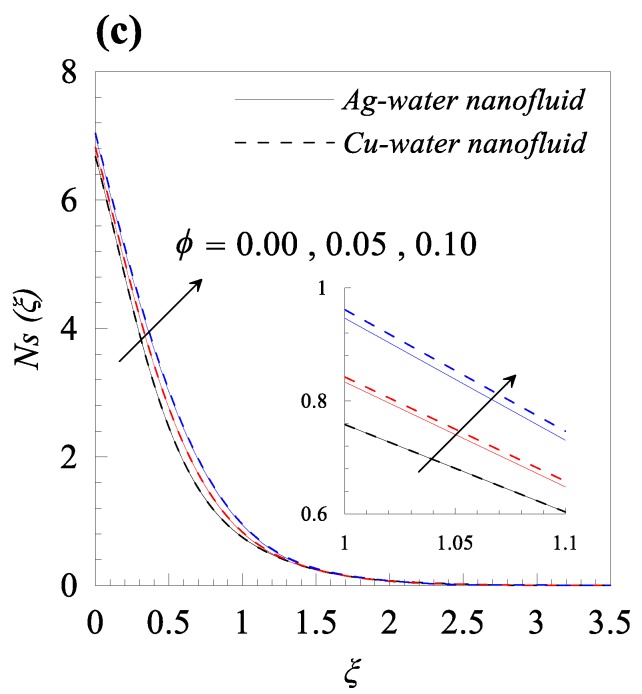
Effects of nanoparticle solid volume fraction *φ* on (**a**) velocity profile *g*’(*ξ*), (**b**) temperature distribution *θ*(*ξ*), and (**c**) entropy generation *Ns*(*ξ*), when *κ* = 10, *M* = 0.2, *ε* = 0.2, *Ec* = 0.3, and *λ* = 0.5.

**Figure 5:**
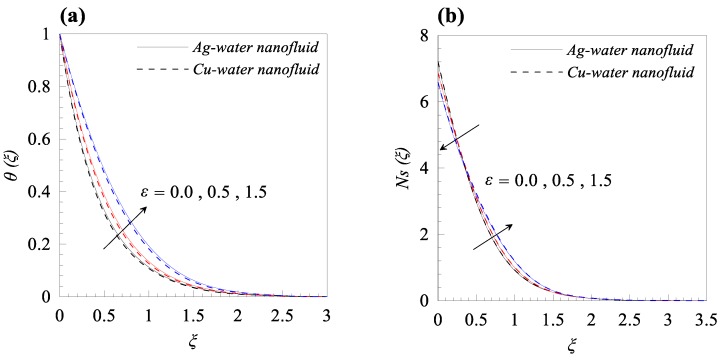
Effects of variable thermal conductivity parameter *ε* on (**a**) temperature distribution *θ*(*ξ*) and (**b**) entropy generation *Ns*(*ξ*) when *κ* = 10, *M* = 0.2, *φ* = 0.01, *Ec* = 0.3, and *λ* = 0.5.

**Figure 6:**
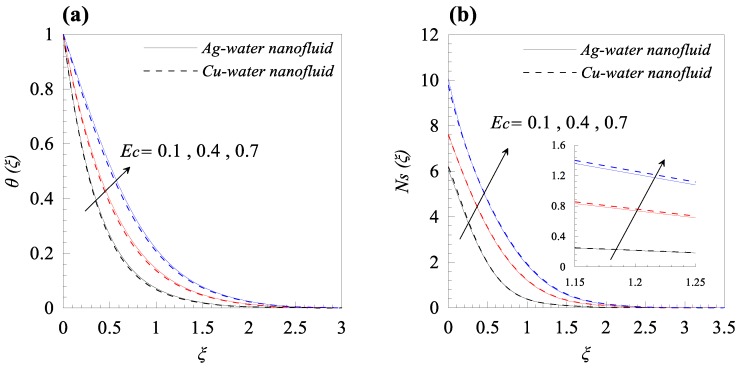
Effects of Eckert number *Ec* on (**a**) temperature distribution *θ*(*ξ*) and (**b**) entropy generation *Ns*(*ξ*), when *κ* = 10, *M* = 0.2, *φ* = 0.01, *ε* = 0.2, and *λ* = 0.5.

**Figure 7:**
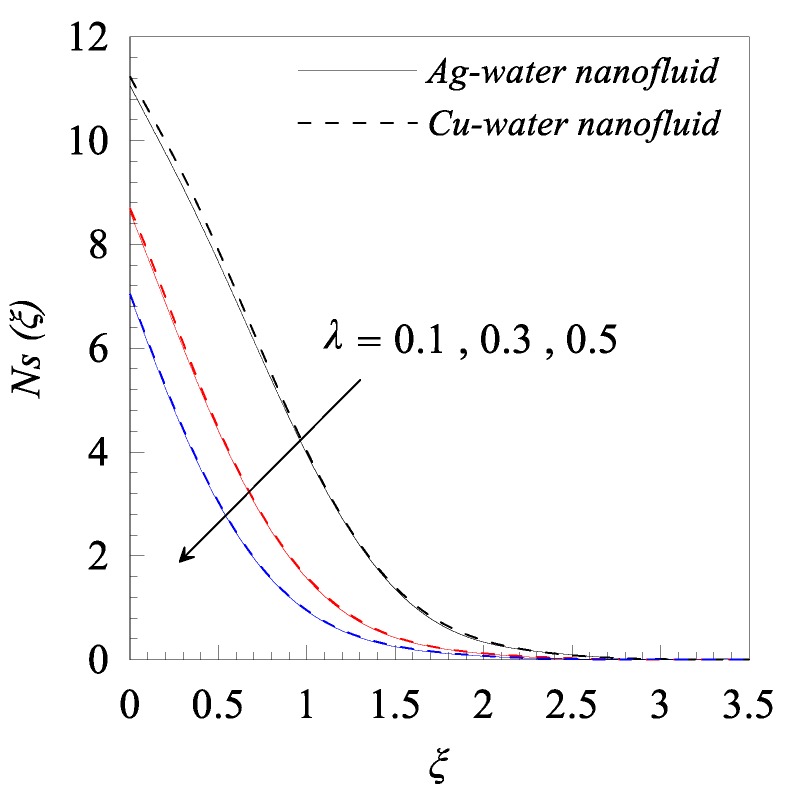
Effects of temperature difference parameter *λ* on entropy generation *Ns*(*ξ*) when *κ* = 10, *M* = 0.2, *φ* = 0.01, *ε* = 0.2, and *Ec* = 0.3.

From Figure 2a, we observed that rise in the dimensionless radius of curvature reduces the velocity profile $g^{\prime }\left(\xi \right)$ of both nanofluids, i.e., Cu–water and Ag–water. Further, for fixed values of κ, the fluid velocity vanished asymptotically. In addition, it was also found that the nanofluid containing Ag nanoparticles flow with less velocity as compared to the nanofluid containing Cu nanoparticles, and this is because of the high density of Ag nanoparticles. Figure 2b describes the influence of dimensionless radius of curvature κ on temperature distribution θ(ξ). The reduction in temperature θ(ξ) was observed with increasing κ, i.e., with decreasing the bending of the curved surface. Furthermore, for the fixed value of *κ*, Cu–water nanofluid has thinner thermal boundary layer as compared to Ag–water nanofluid. It was observed that entropy generation number *N_s_* has an inverse relation with *κ*, as shown in Figure 2c. Further, it was also observed that more entropy was generated in Cu–water nanofluid as compared to Ag–water nanofluid. Additionally, less entropy was generated in the flow past over the flat surface, as compared to the flow over a curved surface for both type of nanofluids. A resistive force known as Lorentz force was generated, due to the applied magnetic field and, as a result, the nanofluid motion decelerated with increasing values of magnetic parameter *M*, as shown in Figure 3a. In addition, the velocity boundary layer thickness is thin for Ag–water nanofluid in comparison with Cu–water nanofluid. The applied magnetic field caused an induced current and, due to the flow of the induced current, heat was dissipated (Ohmic heating), which led to a rise in the temperature of the nanofluid, as depicted in Figure 3b. Due to the high thermal conductivity of Ag gnanoparticles, the thickness of thermal boundary layer of Ag–water is thicker than that of Cu–water nanofluid. The effects of the increasing values of magnetic parameter *M* on entropy generation *N_s_* is represented in Figure 3c. The plot illustrates that *N_s_* is the increasing function of *M* at the surface of curved boundary and its vicinity. Further, after a certain value of the similarity variable *ξ*, the opposite effects are observed. An increase in the solid volume fraction of nanoparticle *φ* leads to an increase in the density of nanofluid and, as a result, the velocity of nanofluid decreases as shown in Figure 4a. The significant influence of *φ* on temperature distribution *θ*(*ξ*) is exhibited graphically in Figure 4b, from which we found that as increases, *θ*(*ξ*) increases. Physically, an increase in *φ* led to a rise in the thermal conductivity of nanofluid and, consequently, an increase in the temperature of fluid. For fixed non-zero values of *φ*, the temperature of nanofluid containing silver nanoparticles was higher than that of containing copper nanoparticles. An increase in entropy generation was noticed with rising values of solid volume fraction of nanoparticles. Comparative analysis revealed that the entropy production in the flow of nanofluid containing copper nanoparticles was greater than that of containing silver nanoparticles. The temperature field *θ*(*ξ*) was enhanced with the increasing values of variable thermal conductivity parameter *ε* for both cases, as shown in Figure 5a. It was observed from Figure 5b that *N_s_* reduces at the surface of stretching boundary, and its vicinity with increasing values of *ε*. An opposite effect was found after a certain value of *ξ* for both type of nanofluids. Figure 6a exhibits the influence of Eckert number *E_c_* on temperature distribution *θ*(*ξ*). It was evident from the plot that *θ*(*ξ*) rises with an increase in Eckert number. This happens because the friction between the fluid layers enhances with the rising values of *E_c_*, and this led to an increase in the temperature of both types of nanofluids. The variation of entropy generation *N_s_* with rising values of Eckert number *E_c_* is elucidated in Figure 6b. From this plot, we found that *N_s_* enhances with amplifying values of Eckert number. This happens because of the dissipative nature of frictional force between the fluid layers. Further, the effects were more prominent at the surface of curved boundary, and this is because of the high thermal and velocity gradients at the boundary. Figure 7 was made to examine the influence of temperature difference parameter *λ* on entropy generation *N_s_*. From this plot, it is found that *N_s_* decreases with the rising values of *λ*. Less entropy is generated in the flow of Ag–water nanofluid as compared to Cu–water nanofluid. Further, this plot suggests that entropy generation can be minimized by reducing the operating temperature (*T_w_* − *T_b_*).

Table 6 shows the variation in local skin friction coefficient −Res0.5Cfs and Nusselt number Res−0.5Nus with the increasing values of *φ*, *ε*, *κ*, *M*, and *Ec*. The rate of increase/decrease in −Res0.5Cfs and Res−0.5Nus is estimated by using the linear regression model. The positive sign of the slope shows that −Res0.5Cfs is an increasing function of *φ* and *M* for both cases. Further, it was found that the rate of increase in −Res0.5Cfs dominates for Ag–water nanofluid with rising values of *φ*. The rate of increase in −Res0.5Cfs, with rising values of *M*, was found to be greater for Cu–water as compared to Ag–water nanofluid. The zero slope shows that −Res0.5Cfs does not change with increasing values of variable thermal conductivity parameter *ε* and Eckert number *Ec* From the sign of the slope, it is evident that Res−0.5Nus increases with increasing values of *φ* and *κ*. Furthermore, it was also observed that Res−0.5Nus is a decreasing function of *ε*, *M*, and *Ec*.

## 6. Conclusions

Heat transfer analysis and entropy generation in the flow of Ag–water and Cu–water nanofluid over a curved stretching sheet was studied. In the present model, frictional heating effect was considered in the energy equation. In addition, the thermal conductivity was assumed to be temperature dependent and this led to increases in the nonlinearity of the governing equations. Consequently, it was not possible to obtain the exact closed form solutions. Therefore, we obtained numerical solutions using the Chebyshev–Gauss–Lobatto spectral method (CGLSM). The following are the main outcomes of the present study:

- The enhancement in dimensionless radius of curvature *κ* (reducing bending of the curved sheet), solid volume fraction of nanoparticles *φ*, and magnetic parameter reduced the velocity of both types of nanofluids. Furthermore, velocity dominated for Cu–water nanofluid.
- A rise in temperature was observed with increasing values of magnetic parameter *M*, solid volume fraction of nanoparticles *φ*, variable thermal conductivity parameter *ε*, and Ecker number *Ec*. Moreover, the temperature inside the boundary layer containing silver nanoparticles was high, as compared to copper nanoparticles.
- Decrement in the temperature distribution θ(ξ) was observed with decreasing bending in the curved surface (i.e., increasing *κ*).
- The thermal boundary layer thickness dominated for Ag–water nanofluid due to high effective thermal conductivity.
- One can reduce the entropy generation *Ns* by decreasing the operating temperature difference (*T_w_* − *T_b_*) and curvature of the curved boundary (i.e., by increasing the dimensionless radius of curvature *κ*).
- Entropy generation *Ns* was enhanced with rising values of Eckert number *Ec* and magnetic parameter *M*.

## Figures and Tables

**Table 1:** Thermophysical properties of some nanoparticles and water [21,49,50].

Properties	Base Fluid (Water)	Ag (Silver)	Cu (Copper)
$c_{p}\left(J/kgK\right)$	4179	235	385
k(W/mK)	0.613	429	401
$\rho \left(kg/m^{3}\right)$	997.1	10,500	8933
$\sigma \left(S\cdot m^{-1}\right)$	5.5 × 10^−6^	6.3 × 10^7^	5.96 × 10^7^
Pr	6.8	-	-

**Table 2:** Validation of our numerical results obtained by Chebyshev–Gauss–Lobatto spectral method (CGLSM) for −Res0.5Cfs with those of the closed form exact solution, in the case where ε=ϕ=0 and κ=∞, when N=100.

M	$\xi_{\infty }$	Present Numerical Results	Present Exact Results
0	21	1.0000000000	1.0000000000
0.25	35	1.1180339887	1.1180339887
1	24	1.4142135623	1.4142135623
2.25	38	1.8027756377	1.8027756377
5	18	2.4494897427	2.4494897427
10	24	3.3166247903	3.3166247903
50	20	7.1414284285	7.1414284285
100	9	10.0498756210	10.0498756211
500	8	22.3830292855	22.3830292856
1000	6	31.6385840390	31.6385840391

**Table 3:** Validation of our numerical results obtained by CGLSM for Res−0.5Nus with those of the closed form exact solution, in the case where ε=ϕ=0 and κ=∞, when N=100.

Ec	Pr	M	$\xi_{\infty }$	Present Numerical Results	Present Exact Results
0.0	7.0	0.5	14	3.9133020001	3.9133020001
0.3			15	3.1448005650	3.1448005650
0.5			15	2.6324662749	2.6324662749
0.7			16	2.1201319848	2.1201319848
0.1	0.7	0.2	35	1.0090352173	1.0090352173
	1.0		24	1.2621850959	1.2621850959
	3.0		18	2.3816005234	2.3816005234
	7.0		18	3.7598314882	3.7598314882
0.4	3.0	0.0	26	2.2038900906	2.2038900906
		1.0	21	1.6226583382	1.6226583382
		1.5	21	1.3788073543	1.3788073543
		2.0	18	1.1547005383	1.1547005383

**Table 4:** Multiple comparison results for −Res0.5Cfs, when ϕ=ε=Ec=M=0, $\xi_{\infty }=10$, and N=29.

κ	Present Numerical Results		
	*CGLSM	*GDQM	*RKFM
5	1.1576312	1.1576312	1.1576312
10	1.0734886	1.0734886	1.0734886
20	1.0356098	1.0356098	1.0356098
30	1.0235310	1.0235310	1.0235310
40	1.0175866	1.0175866	1.0175866
50	1.0140492	1.0140492	1.0140492
100	1.0070384	1.0070384	1.0070384
200	1.0035641	1.0035641	1.0035641
1000	1.0007993	1.0007993	1.0007993

*CGLSM: Chebyshev–Gauss–Lobatto spectral method. *GDQM: generalized differential quadrature method. *RKFM: Runge–Kutta–Fehlberg method.

**Table 5:** Comparison of our numerical values for −Res0.5Cfs with existing results for different values of κ, when ϕ=ε=Ec=M=0.

κ	Rosca and Pop [11]	Present Results
5	1.15076	1.1576312
10	1.07172	1.0734886
20	1.03501	1.0356098
30	1.02315	1.0235310
40	1.01729	1.0175866
50	1.01380	1.0140492
100	1.00687	1.0070384
200	1.00342	1.0035641
1000	1.00068	1.0007993

**Table 6:** Impact of the emerging parameters *φ*, *ε*, *κ*, *M*, and *Ec* on the engineering quantities −Res0.5Cfs and Res−0.5Nus given by CGLSM for *Ag*–water and *Cu*–water nanofluids, when *ξ*_∞_ = 10 and *N* = 70.

*φ*	*ε*	*κ*	*M*	*Ec*	Ag–Water Nanofluid		Cu–Water Nanofluid	
					−Res0.5Cfs	Res−0.5Nus	−Res0.5Cfs	Res−0.5Nus
0.00	0.2	10	0.2	0.3	1.1846573	1.2160859	1.1846573	1.2160859
0.05					1.4906065	1.4464253	1.4590642	1.4461851
0.10					1.8083488	1.6262670	1.7485023	1.6414604
**Slope (Linear Regression)**					**6.2369150**	**4.1018110**	**5.6384500**	**4.2537450**
0.10	0.0	10	0.2	0.3	1.8083488	3.1662365	1.7485023	3.2955617
	0.5				1.8083488	0.1863395	1.7485023	0.1001680
	1.5				1.8083488	−1.8979797	1.7485023	−2.1188446
**Slope (Linear Regression)**					**0.0000000**	**−3.1915977**	**0.0000000**	**−3.4109482**
0.10	0.2	5	0.2	0.3	1.9290412	1.6103249	1.8713011	1.6288812
		15			1.7708298	1.6303497	1.7104317	1.6445620
		1000			1.7006379	1.6369517	1.6393829	1.6494311
**Slope (Linear Regression)**					**−0.0001520**	**0.0000169**	**−0.0001542**	**0.0000129**
0.10	0.2	10	0.0	0.3	1.6874096	1.6522005	1.6222921	1.6601904
			0.5		1.9707792	1.5674102	1.9166084	1.5918967
			1.5		2.4186108	1.2500458	2.3753224	1.2990534
**Slope (Linear Regression)**					**0.4818052**	**−0.2751404**	**0.4958336**	**−0.2481987**
0.1	0.2	10	0.2	0.1	1.8083488	1.6617211	1.7485023	1.6571309
				0.4	1.8083488	1.5613071	1.7485023	1.5909710
				0.7	1.8083488	1.1758866	1.7485023	1.2674579
**Slope (Linear Regression)**					**0.0000000**	**−0.8097241**	**0.0000000**	**−0.6494550**
